# The Structural Diversity of Carbohydrate Antigens of Selected Gram-Negative Marine Bacteria

**DOI:** 10.3390/md9101914

**Published:** 2011-10-14

**Authors:** Evgeny L. Nazarenko, Russell J. Crawford, Elena P. Ivanova

**Affiliations:** 1 Pacific Institute of Bioorganic Chemistry, Far East Branch of the Russian Academy of Sciences, Vladivostok 690022, Russia; E-Mail: elnaz@piboc.dvo.ru; 2 Faculty of Life and Social Sciences, Swinburne University of Technology, PO Box 218, Hawthorn, Victoria 3122, Australia; E-Mail: rcrawford@swin.edu.au

**Keywords:** carbohydrate antigens, *O*-specific polysaccharides, marine microorganisms

## Abstract

Marine microorganisms have evolved for millions of years to survive in the environments characterized by one or more extreme physical or chemical parameters, e.g., high pressure, low temperature or high salinity. Marine bacteria have the ability to produce a range of biologically active molecules, such as antibiotics, toxins and antitoxins, antitumor and antimicrobial agents, and as a result, they have been a topic of research interest for many years. Among these biologically active molecules, the carbohydrate antigens, lipopolysaccharides (LPSs, *O*-antigens) found in cell walls of Gram-negative marine bacteria, show great potential as candidates in the development of drugs to prevent septic shock due to their low virulence. The structural diversity of LPSs is thought to be a reflection of the ability for these bacteria to adapt to an array of habitats, protecting the cell from being compromised by exposure to harsh environmental stress factors. Over the last few years, the variety of structures of core oligosaccharides and *O*-specific polysaccharides from LPSs of marine microrganisms has been discovered. In this review, we discuss the most recently encountered structures that have been identified from bacteria belonging to the genera *Aeromonas*, *Alteromonas*, *Idiomarina*, *Microbulbifer*, *Pseudoalteromonas*, *Plesiomonas* and *Shewanella* of the *Gammaproteobacteria* phylum; *Sulfitobacter* and *Loktanella* of the *Alphaproteobactera* phylum and to the genera *Arenibacter*, *Cellulophaga*, *Chryseobacterium*, *Flavobacterium*, *Flexibacter* of the *Cytophaga*-*Flavobacterium*-*Bacteroides* phylum. Particular attention is paid to the particular chemical features of the LPSs, such as the monosaccharide type, non-sugar substituents and phosphate groups, together with some of the typifying traits of LPSs obtained from marine bacteria. A possible correlation is then made between such features and the environmental adaptations undertaken by marine bacteria.

## 1. Introduction

Gram-negative bacteria are ubiquitous in marine environments. As with other microorganisms present in sea habitats, they represent an interesting taxonomic lineage, and are a valuable source of natural biologically active substances [1–4]. These substances comprise a wide range of antibiotics, toxins and antitoxins, antitumor and antimicrobial agents and enzymes with a wide activity spectrum. Recently, the peculiar metabolic pathways of marine bacteria have been the subject of intensive research due to their possible employment in the biological decontamination of polluted sites. Head *et al*. [1] reviewed the processes underlying the degradation of hydrocarbons by marine microorganisms in light of current bioremediation strategies. For example, several species belonging to the genus *Shewanella* have been considered for their great biotechnological potential, due to their capabilty of dissimilatory reduction of a wide range of electron acceptors, including metal oxides (e.g., those of Fe(III) and Mn(IV)) and organic pollutants [2].

The lipopolysaccharides (LPSs) from non-pathogenic bacteria have also been the focus of intensive medical and veterinary research because in many cases bacteria that were not previously considered to be human pathogens were found in infected, immuno-compromised patients. In other cases, bacteria that were pathogenic for other mammals, became pathogens for humans, whether they were immuno-compromised or not, and *vice versa* [3]. At present, considerable attention is being given to the elucidation of the chemical structures of LPSs of Gram-negative marine bacteria.

The most complete form of LPS (*S*-type) has a tripartite structure, in which the *O*-antigenic side-chain (normally consisting of polymerized oligosaccharide units) is attached to the hydrophobic anchor, lipid A, via a connecting (core) oligosaccharide, the inner region of which is typically constructed from 3-deoxy-d-*manno*-oct-2-ulosonic acid (Kdo) and l-*glycero*-d-*manno*-heptose (l,d-Hep). In classical LPS, the backbone of lipid A is a β-1′,6-linked disaccharide of 2-amino-2-deoxy-d-glucose (d-glucosamine, d-GlcN) to which fatty acids, typically 3-hydroxyalkanoic acids, are attached by ester or amide linkages. Both the inner core region and lipid A are commonly carried ionizing groups. Anionic functions are contributed by Kdo, phosphate and, sometimes, hexuronic or other acid residues. The phosphate groups often serve as bridges to an amino alcohol (ethanolamine, Et_3_N) or 4-amino-4-deoxy-l-arabinose (l-Ara4N), while glycosidically-linked amino sugar residues sometimes have a free amino group. It seems clear that the charged groups in LPS, like the polar head groups of phospholipids, are important to the molecular organization and functions of the bacterial outer membrane [4].

All regions of LPS display heterogeneity. For example, products described as *S*-type LPS normally consist of the populations of molecular species with different degrees of polymerization of the *O*-specific side chain, including species with few and/or single repeating unit (also called as semi-rough antigen, *SR*-type), and also contain the species without a side chain, *R*-type) and perhaps even species with an incomplete core oligosaccharide (core-defective *R*-types). In many bacteria, the *O*-specific side chains of the LPS vary widely in structure and composition, giving biological identity to individual strains (serotypes or serovars).

In non-encapsulated Gram-negative bacteria producing *S*-type LPS, the side chains are the dominant, heat-stable surface antigen. Biological specificity is achieved through chemical diversity, by means of the variations in composition and structure for which carbohydrates are pre-eminently suited. In addition to the enormous potential for complexity and diversity already being available, even with common hexoses, the scope of variation in *O*-antigens is often further enhanced by the utilization of less common enantiomers and other stereoisomers, monosaccharides of different chain length (C_5_ to C_10_), ketoses as well as aldoses, structural modifications (e.g., positional and cumulative permutations of deoxy, amino and carboxyl functions; esterification, etherification and amidation), branched monosaccharides and non-carbohydrate residues (e.g., polyols, carboxylic and amino acids) [5].

Bacterial *O*-specific heteropolysaccharides are generally composed of oligosaccharide repeating units. In the biosynthesis of polysaccharides, the so-called “biological” repeating unit is first assembled and then polymerised. In most structural studies, only the “chemical” repeating unit has been determined, whereas the “biological” repeating unit may be any cyclic permutation (rearrangement) of that structure. A number of reviews have dealt with the structures of bacterial carbohydrate antigens e.g., those by Kenne and Lindberg [6], Knirel and Kochetkov [7], Jansson [8], Knirel [9].

The *O*-specific polysaccharides (OPSs) obtained from marine bacteria are often anionic in nature. This has been related to their process of adaptation to the marine environment, since the availability of negatively charged sites on the polysaccharide chains creates a suitable site for the formation of ionic interactions mediated by divalent cations. These bridges strengthen the overall packing of the membrane, thus providing further stability towards external stressors as high pressure. Polysaccharides obtained from marine bacteria have been previously reviewed [10–12].

The OPS is covalently attached to the core oligosaccharide. This region of the LPS shows lower intra-species variability, and is characterized by the presence, in the inner region, of typical monosaccharides, namely 3-deoxy-d-*manno*-oct-2-ulosonic acid (Kdo) and l-*glycero*-d-*manno*-heptose (l,d-Hep) [13]. In the outer core region, neutral or acidic monosaccharides, as well as 2-deoxy-2-aminosugars, are typically encountered. In marine bacteria, archetypal chemical features of the core region have also been encountered, such as the replacement of Kdo by its 8-deoxy-8-amino analogue (Kdo8N) in *Shewanella* [14–16], the occurrence of the d-*glycero*-d-*manno*-heptose (d,d-Hep) [15,16], and the occurrence of monosaccharides connected via phosphodiester bonds to the linear backbone of the oligosaccharide, as observed in the core region of the LPS from *Arenibacter certesii* [17] and *Alteromonas addita* [18]. Furthermore, as for the OPSs, core oligosaccharides from marine bacteria often show a remarkable negative charge density, inferred by the presence of a great number of phosphate substituents and/or acidic monosaccharides.

The methods leading to the structural characterization of LPSs and lipooligosaccharides (LOSs) include a complex series of extraction, purification and degradation steps. These are obviously supported by an extensive succession of chemical analyses, mainly based on chemical derivatization and gas chromatography-mass spectrometry (GC-MS) analyses, in order to achieve the complete definition of the monosaccharide and lipid content, and completed by matrix-assisted laser desorption/ionization mass spectrometry (MALDI-MS) and high resolution nuclear magnetic resonance spectroscopy (NMR), both of which allow the full structural description of the sub-domains composing the LPS structure.

The chemical structures are determined using mainly sugar and methylation analyses, ^1^H and ^13^C NMR spectroscopy, including 2D NMR experiments, homo- and heteronuclear correlation spectroscopy such as homonuclear ^1^H,^1^H correlation spectroscopy (COSY), total correlation spectroscopy (TOCSY), heteronuclear H-detected multuquantum and multi bond correlation (^1^H,^13^C HMQC and HMBC), nuclear Overhauser effect (NOE) spectroscopy (one- and two-dimensional 1D NOE, 2D nuclear Overhauser effect spectroscopy NOESY and rotational Overhauser effect spectroscopy ROESY), and by the attached proton test (APT).

In this review, we consider the chemical composition and structure of *O*-antigens, as well as LPS core oligosaccharides that are integral components of the cell wall surfaces of some Gram-negative marine bacteria. These bacteria are abundant in the marine environment inhabiting coastal, deep-sea and high sea areas, hydrothermal vents and bottom sediments, marine invertebrates and animals.

## 2. Structure of Carbohydrate Antigens of *Gammaproteobacteria*

### 2.1. Genus *Alteromonas*

This genus belongs to Alteromonadaceae family and comprises 13 validly described species [19,20]. To date, the structural investigation of the LPS of three species of the genus *Alteromonas* have been completed. Two structures of the *R*-LPSs from *Alteromonas addita* KMM 3600^T^, *Alteromonas macleodii* ATCC 27126^T^ have been reviewed by Leone *et al.* [12], and were found to be an extremely short oligosaccharide chain with a high negative charge density due to the occurrence of 3-deoxy-d-*manno*-oct-2-ulosonic acid (Kdo) and phosphate groups.

The *O*-specific polysaccharide from the *S*-type LPS of *Alteromonas addita* KMM 3600^T^ [21,22] is comprised of trisaccharide repeating units containing l-rhamnose, d-glucose, and d-galactose residues. It was established that the *O*-specific polysaccharide consists of linear trisaccharide repeating units having the following structure (1) [23]:

(1) →3)-α-D-Galp-(1→3)-α-L-Rhap-(1→3)-α-D-Glcp-(1→

The structure of the highly acidic extracellular polysaccharide produced by the mesophilic species, “*Alteromonas infernus*” [24], found in deep-sea hydrothermal vents and grown under laboratory conditions, has been investigated using partial depolymerization, methylation analysis, mass spectrometry and NMR spectroscopy. The repeating unit of this polysaccharide is a nonasaccharide with the following structure (2) [25]:

(2)
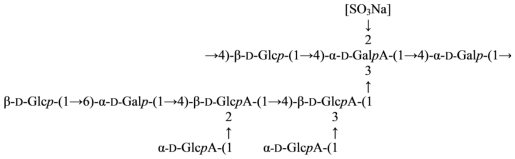


### 2.2. Genus *Microbulbifer*

The genus *Microbulbifer* was proposed by González *et al.* (1997) to accommodate a novel rod-shaped and strictly aerobic marine bacterium belonging to the *Gammaproteobacteria*. Isolates belonging to this genus have been isolated from a marine enrichment community growing on pulp-mill effluent, and the original inoculum was obtained from a salt marsh on the coast of Georgia, USA [26]. At present the genus consists of 14 validly described species isolated from various marine sources [19]. The composition and structure of carbohydrate-containing biopolymers from the cell membrane of this genus species have not been studied.

The capsular polysaccharide (CPS) containing d-galactosamine uronic acid and d-alanine residues were isolated from the culture of *Microbulbifer* sp. KMM 6242 [27]. The combined chemical and NMR analyses showed that the CPS is a homopolymer of 2-acetamido-2-deoxy-*N*-(d-galacturonyl)-d-alanine of the following structure (3):

(3) →4)-β-D-GalpNAcA6(D-Ala)-(1→

Such an amide of d-galactosamine uronic acid was found in bacterial exopolysaccharides for the first time.

An *O*-specific polysaccharide containing d-ribose and d-galactose residues was isolated from the cell-membrane lipopolysaccharide. On the basis of sugar analysis and NMR spectroscopy data the following structure of the disaccharide repeating unit of the polysaccharide was established (4):

(4) →3)-β-D-Ribf-(1→4)-β-D-Galp-(1→

### 2.3. Genus *Pseudoalteromonas*

Gram-negative bacteria of the genus *Pseudoalteromonas* are aerobic non-fermentative prokaryotes. They are widespread obligatory marine microorganisms and require seawater based culture media for their growth. The bacteria produce a wide range of biologically active compounds, such as antibiotics, toxins and antitoxins, antitumor and antimicrobial agents, as well as enzymes with a wide spectrum of action.

Common features of most polysaccharides of the genus *Pseudoalteromonas* are their acidic character and the presence of unusual sugars and non-sugar substituents with the absence of any structural similarity of the repeating units. For example, l-iduronic acid [28], 2-acetamido-2-deoxyhexuronic acids with the d-*galacto* [29,30], l-*galacto* [29,31] and l-*gulo* [32] configurations, 3-deoxy-d-*manno*oct- 2-ulosonic acid [33,34], 5-acetamido-3,5,7,9-tetradeoxy-7-formamido-l-*glycero*-l-*manno*-non-2- ulosonic acid [30], 2,3-diacetamido-2,3-dideoxy-d-mannuronoyl-l-alanine [35], *R*-lactic acid [36], sulfate [37,38] and glycerophosphate [39] have been found among uncommon acidic components of the polysaccharides of *Pseudoalteromonas*.

The typical components include various *N*-acyl derivatives of 6-deoxyamino sugars, such as *N*-acetylated 2-amino-2,6-dideoxy-d-glucose (d-quinovosamine) [30], l-galactose (l-fucosamine) [33] and l-talose [33], 3-(*N*-acetyl-d-alanyl)amino-3,6-dideoxy-d-glucose [29], 4-(*N*-acetyl-d-alanyl) amino-4,6-dideoxy-d-glucose [31] and 3,6-dideoxy-3-(4-hydroxybutyramido)-d-galactose [32], as well as *N*-acetyl and *N*-[(*S*)-3-hydroxybutyryl] derivatives of 2,4-diamino-2,4,6-trideoxy-d-glucose (bacillosamine) [28,29,33,35]. In cases where polysaccharides other than *O*-antigens were isolated from encapsulated bacteria, there was no direct evidence that they were constituents of the capsule.

The structure of *O*-specific polysaccharide from lipopolysaccharide of *Pseudoalteromonas marinoglutinosa* KMM 232 (*S*-form) that consists of disaccharide repeating units containing a sulphate group has been described earlier [37]. An unusual structure of acidic *O*-specific polysaccharide was found in one of the recently described species, *Pseudoalteromonas agarivorans* [40], distinct by their stable colony dissociation into *R*- and *S*-form. A distinctive feature of the agarolytic strain KMM 232 is that it forms *R*-type (rough) colonies together with *S*-type (smooth) colonies during its cultivation on solid media. The *R*-form of KMM 232 has traits that are typical characteristics of *R*-bacteria, including *R*-colony formation, loss of flagella, and high sensitivity to antibiotics. The dissociation of *R*- and *S*-forms was observed to be stable for the strain KMM 232 and in other strains of the species *P. agarivorans*. This was the first time *R*- and *S*-form dissociation was described for bacteria of the genus *Pseudoalteromonas* [40].

An acidic *O*-specific polysaccharide containing l-rhamnose, 2-acetamido-2-deoxy-d-galactose, 2,6-dideoxy-2-(*N*-acetyl-l-threonine)amino-d-galactose, and 2-acetamido-2-deoxy-d-mannuronic acid was obtained by mild acid degradation of the lipopolysaccharide of the marine bacterium *Pseudoalteromonas agarivorans* KMM 232 (*R*-form) followed by gel-permeation chromatography. The polysaccharide was subjected to Smith degradation to give a modified polysaccharide with trisaccharide repeating unit containing l-threonine. The initial and modified polysaccharides were studied by sugar analysis and ^1^H- and ^13^C NMR spectroscopy, including COSY, TOCSY, ROESY, and HSQC experiments, and the structure of the branched tetrasaccharide repeating unit of the polysaccharide was established as follows (5) [41]:

(5)
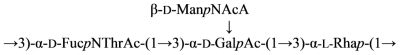


It is noteworthy that one of the polysaccharide components, namely 2,6-dideoxy-2-(*N*-acetyl-lthreonine) amino-d-galactose, has not been found earlier in nature. It is known that *R*-forms of terrestrial bacteria, unlike *S*-forms lose *O*-specific chains of the lipopolysaccharides and thus attain other properties. A feature of the marine bacterium *P. agarivorans* KMM 232 is that its *S*- and *R*-forms also synthesize lipopolysaccharides, in which *O*-specific polysaccharides have various structures. In the given strain the loss of *O*-specific polysaccharides in the *R*-form was not observed and it was suggested that such variability probably reflects the readiness of the bacterium to adapt to environmental changes. Moreover, it can be shown that Gram-negative marine bacteria substantially differ from terrestrial bacteria in their structural organization and the mechanism by which the cell wall functions [41].

A group of pigmented *Pseudoalteromonas* species [42] is known for their ability to synthesize a variety of pigments (prodigiosin-like, carotenoids and some others) and inhibitory (including antifungal) compounds [43]. Among those red-pigmented bacteria, *Pseudoalteromonas rubra* with the type strain NCMB 1890^T^ (=ATCC 29570^T^ were originally isolated from the Mediterranean water off Nice in 1976 by M. Gauthier [44]. The type strain of *P. rubra* has been found to produce an extracellular polyanionic antibiotic that modifies bacterial respiration [45] and cell-bound fatty acids and phospholipids with surface activity [46]. These findings provide evidence of ecophysiological diversification of pseudoalteromonads and on particular remarkable metabolic capacity of *Pseudoalteromonas rubra*, which may play an important role in coexistence and survival of numerous bacterial taxa in marine environments [47].

**Figure 1:**
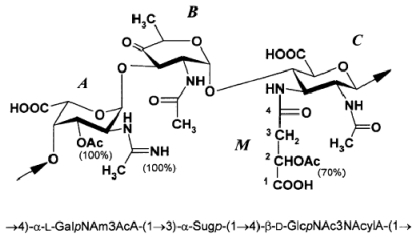
*Pseudoalteromonas rubra* ATCC 29570^T^ [48]. Reprinted with permission from Elsevier. M—malic acid residue.

The structure of the *O*-specific polysaccharide from *P. rubra* type ATCC 29570^T^ has been elucidated using ^1^H and ^13^C NMR spectroscopy, including 2D COSY, TOCSY, gradient pulse phase sensitive (gNOESY), ROESY, ^1^H, ^13^C gradient pulse (gHMQC) and gradient pulse (gHMBC) experiments. It was found that the polysaccharide consisted in 4-keto hexose, 2-acetamido-2,6-dideoxy-d-*xylo*-hexos-4-ulose (Sug, residue B) and two di-*N*-acyl derivatives of uronic acids: 2-acetamidino-3-acetamido-2,3-dideoxy-l-galacturonic acid (residue A) and 2-acetamido-3-(*N*-malyl)amino-2,3-dideoxy-d-glucuronic acid (residue C). The *O*-polysaccharide of *Pseudoalteromonas rubra* ATCC 29570^T^ has the structure shown below (Figure 1) [48]. It contains two rarely occurring components, malic acid and 2-acetamido-2,6-dideoxy-d-*xylo*-hexos-4-ulose (Sug). To the best of our knowledge, d-malic acid has been hitherto identified only once in a polysaccharide from *Shewanella algae* BrY [49].

The same keto sugar has been found earlier as a component of capsular polysaccharides from *Streptococcus pneumonia* type 5 [50] and *Vibrio ordalii* O:2 [51] and an *O*-polysaccharides of *Flavobacterium columnare* ATCC 43622 [52] and *Vibrio vulnificus* clinical isolate YJ016 (6, 7) [53].

(6) →4)-α-L-GalpAc-(1→3)-α-D-Sugp-(1→4)-β-L-GlcpNmalylA(→

where Sug is 2-acetamido-2,6-dideoxy-d-*xylo*-hexos-4-ulose, Am is acetimidoyl and malic acid residue *(M)* is *O*-acetylated in ~70% of the units (7).

(7) →4)-β-D-GlcpNAc3NAcylAN-(1→4)-α-L-GalpNAmA-(1→3)-α-D-QuipNAc-(1→

where QuiNAc stands for 2-acetamido-2,6-dideoxyglucose, GalNAmA for 2-acetimidoylamino-2- deoxygalacturonic acid, GlcNAc3NAcylAN for 2-acetamido-3-acylamino-2,3-dideoxyglucuronamide and acyl for 4-d-malyl (~30%) or 2-*O*-acetyl-4-d-malyl (~70%). The structure of the polysaccharide studied highly resembles that of a marine bacterium *Pseudoalteromonas rubra* ATCC 29570^T^ reinvestigated in this work. The latter differs in: (i) the absolute configuration of malic acid (l *vs.* d); (ii) 3-*O*-acetylation of GalNAmA; and (iii) replacement of QuiNAc with its 4-keto biosynthetic precursor.

The polysaccharides of *Pseudoalteromonas rubra* ATCC 29570^T^ and *V. vulnificus* CECT 5198 and S3-I2-36 are remarkably similar in structure too [54]. It was found that 2,3-diamino-2,3-dideoxy-dglucuronic acid (GlcN3NA) exists as an amide and the malic acid is in the l form. Therefore, the polysaccharide of *P. rubra* ATCC 29570^T^ has the structure shown in Figure 1, which differs from the polysaccharide structure of *V. vulnificus* CECT 5198 and S3-I2-36 in: (i) the absolute configuration of malic acid (l *vs.* d); (ii) 3-*O*-acetylation of GalNAmA; and (iii) replacement of QuiNAc with its biosynthetic precursor, 2-acetamido-2,6-dideoxy-d-*xylo*-hexos-4-ulose. It can be suggested that both bacteria had originally the same sugar synthesis pathway and glycosyl transferase genes but in *Pseudoalteromonas rubra* the gene for reductase that converts the 4-keto sugar into QuiNAc, has been inactivated by a mutation. Surprisingly, in all these polysaccharides, as in the *O*-polysaccharide of *Pseudoalteromonas rubra*, the mentioned above 4-keto sugar is (1→4)-linked to a β-d-*gluco*-configurated monosaccharides and glycosylated at position 3 by a monosaccharide having the α-l-configuration. When obtained from non-bacterial sources, such keto sugar residues were found to be a component of some saponins from starfish [55–57].

### 2.4. Genus *Plesiomonas*

The genus *Plesiomonas* belongs to Enterobacteriaceae family and consists of only one species—*Plesiomonas shigelloides.* Bacteria of this species (previously *Aeromonas shigelloides*) is a ubiquitous, facultatively anaerobic, flagellated, Gram-negative, rod-shaped bacterium which has been isolated from a variety of sources such as freshwater, surface water and many wild and domestic animals, and is particularly common in tropical and subtropical habitats [58].

DNA-DNA hybridization tests showed that all *Plesiomonas shigelloides* strains are closely related to each other thus constituting a separate well defined genus within the family Vibrionaceae. *P. shigelloides* shares biochemical and antigenic properties with Enterobacteriaceae and Vibrionaceae; however, genetically it shows only 8% and 7% similarity, respectively [58]. Infections with *P. shigelloides* have been strongly associated with drinking untreated water [59,60], eating uncooked shellfish or with travel to developing countries [61,62]. Recent studies have suggested that *P. shigelloides* is an opportunistic pathogen in immunocompromized hosts [63] especially neonates [64]. It has been associated with diarrhoeal illness [65] and other diseases in normal hosts as well. *Plesiomonas shigelloides* has been isolated from a variety of clinical specimens including cerebrospinal fluid, wounds and the respiratory tract. Reported cases of meningitis and bacteraemia [64] caused by *P. shigelloides* are of special interest because of their seriousness. *P. shigelloides* causes both localized infections originating from infected wounds and gastrointestinal infections, which can disseminate to other parts of the body [66].

The serotyping scheme of *Plesiomonas shigelloides* proposed by Shimada and Sakazaki [67] and Aldova *et al.* [68] includes 102 *O*-serotypes, some *O*-antigens showing cross-reactivity with antisera directed against lipopolysaccharides (LPS) of *Shigella sonnei*, *Shigella dysenteriae* strains 1, 7 and 8, *Shigella boydi* strains 2, 9 and 13, and *Shigella flexneri* strain 6 [68,69]. Two *P. shigelloides* strains were found to share the structure of *O*-antigens with those of *S. flexneri* and *S. dysenteriae* [70].

Although the antigenic schemes of *P. shigelloides* have been extensively studied with the serological methods mentioned above, the unique structures of *O*-specific polysaccharides are known only for a few strains [70].

Sugar and methylation analyses of native polysaccharides together with one-dimensional ^1^H and ^13^C NMR spectroscopy revealed that the two polysaccharides from strains 22074 and 12254 of *P. shigelloides* are identical. The structure of the polysaccharide from strain 22074 was deduced from uronic acid degradation and by NMR spectroscopy where heteronuclear multiple bond connectivity and two-dimensional nuclear Overhauser effect spectroscopy experiments established the pentasaccharide repeating unit as (8):

(8) →4)-α-D-GalpA-(1→3)-α-D-GlcpNAc-α-L-Rhap-(1→2)-α-L-Rhap-(1→2)-α-L-Rhap-(1→

The comparison *O*-PS structure from strains 22074 and 12254 of *Plesiomonas shigelloides* showed the both strains contains a mixture of antigens specific for S. *flexneri* 6 and the common group antigen of *S. flexneri* species and *S. dysenteriae* 1 (see below). This explains the basis of cross-reactivity. Moreover, *P. shigelloides* strains exhibit moderate invasion of Hep-2 cells [69] which suggest that they may cause limited invasion of intestinal epithelial cells, and this is a desirable attribute of a vaccine strain against *Shigella* infection [69]. Since they also share antigens with two major species of *Shigella*, these strains have the potential to give broad protection against shigellosis if used as vaccine strains (9); Structures of the *O*-antigens from *Shigella*, cross-reacting with strains 22074 and 12254 of *Plesiomonas shigelloides.*

(9) $$\begin{array}{c} \to 2)\text{-}\alpha \text{-L-Rha}p\text{-}(1\to 2)\text{-}\alpha \text{-L-Rha}p\text{-}(1\to 3)\text{-}\alpha \text{-L-Rha}p\text{-}(1\to 3)\text{-}\beta \text{-D-Glc}p\text{NAc-}(1\to \\ \mathrm{Shigella}\;\mathrm{flexneri}\;Y\\ \to 2)\text{-}\alpha \text{-L-Rha}p\text{-}(1\to 2)\text{-}\alpha \text{-L-Rha}p\text{-}(1\to 4)\text{-}\beta \text{-D-Gal}p\text{A-}(1\to 3)\text{-}\beta \text{-D-Gal}p\text{NAc-}(1\to \\ \mathrm{Shigella}\;\mathrm{flexneri}\;6\\ \to 3)\text{-}\alpha \text{-L-Rha}p\text{-}(1\to 3)\text{-}\alpha \text{-L-Rha}p\text{-}(1\to 2)\text{-}\alpha \text{-D-Gal}p\text{-}(1\to 3)\text{-}\alpha \text{-D-Glc}p\text{NAc-}(1\to \\ \mathrm{Shigella}\;\mathrm{dysenteriae}\;1 \end{array}$$

The structure of the *O*-specific side chain of the lipopolysaccharide (LPS) of *P. shigelloides* O54, strain CNCTC 113/92 has been investigated by NMR spectroscopy, matrix-assisted laser desorption/ionization time of flight mass spectrometry and sugar and methylation analysis. It was concluded that the polysaccharide is composed of a hexasaccharide repeating unit as follows (10) [71]:

(10)
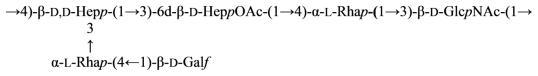


where β-d,d-Hep*p* is β-d-*glycero*-d-*manno*-heptopyranose and 6d-β-d-Hep*p* is 6-deoxy-β-d-*manno*heptopyranose. This structure represents a novel hexasaccharide repeating unit of bacterial *O*-antigen that is characteristic and unique to the *P. shigelloides* strain.

The lipopolysaccharide of *Plesiomonas shigelloides* serotype O74:H5 (strain CNCTC 144/92) was obtained with the hot phenol/water method, but unlike most of the S-type enterobacterial lipopolysaccharides, the *O*-antigens were preferentially extracted into the phenol phase. The poly- and oligosaccharides released by mild acidic hydrolysis of the lipopolysaccharide from both phenol and water phases were separated and investigated by ^1^H and ^13^C NMR spectroscopy, matrix-assisted laser-desorption/ionization time-offlight MS (MALDI-TOF) mass spectrometry, and sugar and methylation analysis. The *O*-specific polysaccharide and oligosaccharides consisting of the core, the core with one repeating unit, and the core with two repeating units were isolated. It was concluded that the *O*-specific polysaccharide is composed of a trisaccharide repeating unit with the structure (11) [72]:

(11) →2)-β-D-Quip3NAcyl-(1→3)-α-L-Rhap2OAc-(1→3)-α-D-FucpNAc-(1→

in which d-Qui3NAcyl is 3-amino-3,6-dideoxy-d-glucose acylated with 3-hydroxy-2,3-dimethyl-5- oxopyrrolidine-2-carboxylic acid. The major oligosaccharide consisted of a single repeating unit and a core oligosaccharide. This undecasaccharide contains information about the biological repeating unit and the type and position of the linkage between the *O*-specific chain and core. The presence of a terminal [β-d-Qui*p*3NAcyl-(1→] residue and the [→3)-β-d-Fuc*p*NAc-(1→4)-α-d-Gal*p*A] element showed the structure of the biological repeating unit of the *O*-antigen and the substitution position to the core. The [→3)-β-d-Fuc*p*NAc-(1→] residue has the anomeric configuration inverted compared to the same residue in the repeating unit. The core oligosaccharide was composed of a nonphosphorylated octasaccharide, which represents a novel core type of *P. shigelloides* LPS characteristic of serotype O74. The similarity between the isolated *O*-specific polysaccharide and that found on intact bacterial cells and lipopolysaccharide was confirmed by high resolution-magic angle spin (HR-MAS) NMR experiments.

The structure of the *O*-chain of the lipopolysaccharide (LPS) from *P. shigelloides* strain 302-73 (serotype O1) was determined by chemical analysis, 1D and 2D NMR spectroscopy and MALDI-TOF mass spectrometry. The polysaccharide was constituted by an inear pentasaccharidic repeating unit as follows (12) [73]:

(12) $$\begin{array}{c} \to 3)\text{-}\alpha \text{-L-PneNAc}4\text{OAc-}(1\to 4)\text{-}\alpha \text{-L-Fuc}p\text{NAc-}(1\to 4)\text{-}\alpha \text{-L-Fuc}p\text{NAc-}(1\to 4)\text{-}\alpha \text{-L-Fuc}p\text{NAc-}(1\to \\ \text{D-QuiNAc}4\text{Nb-}(1\to \end{array}$$

(PneNAc = 2-acetamido-2,6-dideoxy-talose, Hb = *S*-3-hydroxybutanoyl. PneNAc *O*-acetylation was not stoichiometric and was found to be about 75%. The position of the *O*-acetyl group and the amount of acetylation were deduced by NMR spectroscopic analysis. All the monosaccharides included in the repeating unit were deoxyamino sugars, which most probably, together with the presence of *O*-acetyl groups, were responsible for the recovery of the LPS in the phenol layer of the phenol/water extract of dried bacteria cells.

*P. shigelloides* strain CNCTC 110/92 (O51) was identified as a new example of plesiomonads synthesizing lipopolysaccharides (LPSs) that show preference for a non-aqueous surrounding during phenol/water extraction. Chemical analyses combined with ^1^H and ^13^C NMR spectroscopy, MALDI-TOF and ESI mass spectrometry showed that the repeating units of the *O*-specific polysaccharides isolated from phenol and water phase LPSs of *P. shigelloides* O51 have the same structure (13) [74]:

(13) →4)-β-D-Glcp-NAc3NRA-(1→4)-α-L-FucpAm3OAc-(1→3)-α-D-QuipNAc-(1→

containing the rare sugar constituent 2,3-diamino-2,3-dideoxyglucuronic acid (GlcpNAc3NRA), and substituents such as d-3-hydroxybutyric acid (R) and acetamidino group (Am). The HR-MAS NMR spectra obtained for the isolated LPSs and directly on bacteria indicated that the *O*-acetylation pattern was consistent throughout the entire preparation. The ^1^H chemical shift values of the structure reporter groups identified in the isolated *O*-antigens matched those present in bacteria. It was found that the *O*-antigens recovered from the phenol phase showed a higher degree of polymerization than those isolated from the water phase. A similar behavior of LPS molecules was previously reported for other strains whose LPS was isolated from both phenol and water phases [75]. Therefore the composition of the *O*-repeating units does not seem to be the only factor influencing the physicochemical properties of such LPSs and suggested that the main solubility factor might be conformational rather than compositional.

The lack of structural data concerning a suitable number of *O*-antigen structures prevented us from deducing anything about the structure–activity relationship. However, it is tempting to speculate that the occurrence of deoxy sugars with hydrophobic substituents in all of the *O*-chain structures so far characterized could suggest a method adopted by *P. shigelloides* to adhere to host cells in aqueous environment. Structure determination is the first step into the understanding of the unusual physicochemical properties of LPSs. The possible role of the LPSs associated with an increased hydrophobicity in the pathogenicity of *P. shigelloides* has not been investigated so far. The data herein presented could be used in the future to study the role of such structures for the type of aggregates formed in an aqueous environment and the biological activity of *P. shigelloides* endotoxin.

The core oligosaccha ride is an important contributor in determining the biological and physical properties of the overall lipopolysaccharide and plays a significant role in interactions with the host.

The core oligosaccharide is relatively conserved in its structure and composition compared to the *O*-chain and can be divided into inner and outer subdomains. The inner core includes unique residues, such as 3-deoxy-d-*manno*-oct-2-ulosonic acid (Kdo) and l-*glycero*-d-*manno*-heptose (Hep) that are characteristic to the LPS. The Kdo residue is located at the reducing end of the oligosaccharide chain and has proven to be critical to the LPS biological activity [76]. At the *O*-4 position of Kdo there may be one or two Kdo glycosyl residues.

Mass spectrometric studies are now playing a leading role in the elucidation of lipopolysaccharide (LPS) structures through the characterization of antigenic polysaccharides, core oligosaccharides and lipid A components including LPS genetic modifications. The conventional MS and MS/MS analyses together with collision induced dissociation (CID) fragmentation provide additional structural information complementary to the previous analytical experiments, and thus contribute to an integrated strategy for the simultaneous characterization and correct sequencing of the carbohydrate moiety [77].

The structure of the core oligosaccharide moiety of the lipopolysaccharide (LPS) of *Plesiomonas shigelloides* O54 (strain CNCTC 113/92) has been investigated by ^1^H and ^13^C NMR, fast atom bombardment mass spectrometry (FAB-MS), MALDI-TOF, monosaccharide and methylation analysis, and immunological methods. It was concluded that the main core oligosaccharide of this strain is composed of a decasaccharide (14) [78]:

(14)
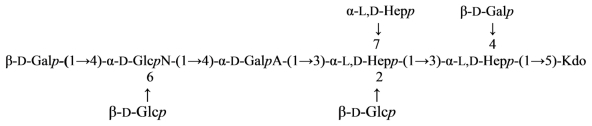


in which α-l,d-Hep*p* is α-l-*glycero*-d-*manno*-heptopyranose. The nonasaccharide variant of the core oligosaccharide (~10%), devoid of β-d-Glc*p* substituting the α-d-Glc*p*N at position 6, was also identified. The core oligosaccharide substituted at position 4 of the outer core β-d-Glc*p* residue with the single *O*-polysaccharide repeating unit was also isolated yielding a hexadecasaccharide structure. The determination of the monosaccharides involved in the linkage between the *O*-specific polysaccharide part and the core, as well as the presence of [→3)-β-d,d-Hep*p*-(1→instead of →3,4)-β-d,d-Hep*p*-(1→] in the repeating unit, revealed the structure of the biological repeating unit of the *O*-antigen. The core oligosaccharides are not substituted by phosphate residues and represent novel core type of bacterial LPS that is characteristic for the *P. shigelloides* serotype O54. Serological screening of 69 different *O*-serotypes of *P. shigelloides* suggests that epitopes similar to the core oligosaccharide of serotype O54 (strain CNCTC 113/92) might also be present in the core region of the serotypes O24 (strain CNCTC 92/89), O37 (strain CNCTC 39/89) and O96 (strain CNCTC 5133) LPS.

The first complete structure of a *Plesiomonas shigelloides* core oligosaccharide has been identified, together with the structure of the biological repeating unit of the *O*-antigen, and the linkage between them. Opinions differ regarding the classification of the genus *Plesiomonas*, because it has some characteristics in common with both Enterobacteriaceae and Vibrionaceae families. A comparison of the 5 S rRNA sequences of a number of Enterobacteriaceae and Vibrionaceae shows that *P. shigelloides* is more related to *Proteus mirabilis* and *Proteus vulgaris* than to any other member of Vibrionaceae tested [79].

The core oligosaccharide of *P. shigelloides* contains the following (15) structural element:

(15)
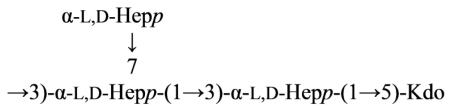


which is present in the majority of characterized enterobacterial and non-enterobacterial core structures.

The LPS of *Plesiomonas shigelloides* serotype O17 is of particular interest since its *O*-antigen structure is identical to that of *Shigella sonnei*, a cause of endemic and epidemic diarrhea and/or dysentery worldwide [80].

The *O*-antigen gene cluster of both *S. sonnei* and *Plesiomonas shigelloides* O17 is located on the plasmid Pinv, apparently acquired by *S. sonnei* from *P. shigelloides* [81,82]. This invasion plasmid is essential for penetration of host epithelial cells and is therefore an important virulence factor [83]. Because of the structural identity of the LPS *O*-specific polysaccharides (*O*-PS) of *S. sonnei* and *Plesiomonas shigelloides* O17, the latter can be used as an immunogenic component of a vaccine against *S. sonnei* [84]. Interpretation of the immunological data and selection of the optimal conjugation conditions require the knowledge of the structure of the LPS core part, which is always present in the O–PS preparations.

*Plesiomonas shigelloides* O17 LPS contains the same *O*-antigenic polysaccharide chain as a causative agent of dysentery, *Shigella sonnei*. This polysaccharide can be used as a component of a vaccine against dysentery. Core part of the *Plesiomonas shigelloides* O17 LPS was studied using NMR and mass spectrometry and the following structure was proposed (16) [85]:

(16)
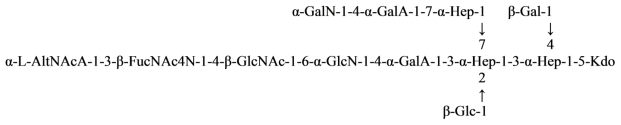


where Hep is l-*glycero*-d-*manno*-heptose and all sugars, except AltNAcA has the d-configuration.

Overall, the structure of the *Plesiomonas shigelloides* O17 core was similar to that of serotype O54 [78]. The difference being the presence of the additional α-GalN-4-α-GalA fragment on *O*-7 of Hep and the absence of the side chain β-Gal at O-4 of GlcN. Attachment of the *O*-chain in serotype O17 was through *O*-4 of β-GlcNAc, whereas in the serotype O54 it was through *O*-4 of β-Glc replacing β-GlcNAc.

The characterization of the core structure from the LPS of the strain 302-73 (serotype O1) was carried out. The LPS obtained after usual PCP (phenol–chloroform–light petroleum) extraction contained a small number of *O*-chain repeating units. The product obtained by hydrazinolysis was analysed by FTICR-ESIMS LPS was hydrolyzed under mild acid conditions and a fraction that contained one *O*-chain repeating unit linked to a Kdo residue was isolated and characterized by FTICR-ESIMS and NMR spectroscopy. Moreover, after an alkaline reductive hydrolysis, a disaccharide α-Kdo-(2→6)-GlcNol was isolated and characterized. The data obtained proved the presence of an α-Kdo in the outer core and allowed the identification of the *O*-antigen biological repeating unit as well as its linkage with the core oligosaccharide (17) [86,87].

The LPS was hydrolyzed under both alkaline and mildly acidic conditions. In both cases, a mixture of oligosaccharides was obtained, which was purified by gel filtration and HPAEC. The oligosaccharides were characterized by chemical analysis, 2D NMR spectroscopy and MALDI-TOF mass spectrometry. A new core structure was found for *P. shigelloides*. In particular, from the analysis of the acid hydrolysed product it was possible to reveal the presence of a of d-*glycero*-d-*talo*-2-octulopyranosonic acid (Ko) residue, which substitutes in part the terminal 3-deoxy-d-*manno*-oct-2-ulosonic acid (Kdo) unit. The Ko residue is not frequently found in core structures.

(17)
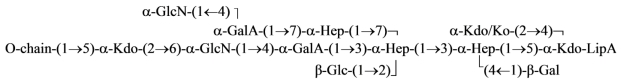


This structure is similar to that of serotype O54 [78] and O17, [85] even if some new features are present in both the inner and outer core. A glucosamine residue was nonstoichiometrically linked to the branching galacturonic acid, and more interestingly, a Ko unit substitutes in part the terminal Kdo residue. The presence of the Ko residue is not frequent in core structures, and to date it has been described as a substitute for Kdo in the LPS of *Yersinia* [88], *Burkholderia* [89], *Acinetobacter* [90], *Serratia* [91]. Finally, this new core oligosaccharide confirmed the lack of a uniform core structure for the unique species of the *Plesiomonas* genus.

### 2.5. Genus *Shewanella*

Bacteria that are currently classified under the generic name *Shewanella* have been the subject of many scientific studies over at least the last 70 years. To date, this rapidly growing genus contains more than 50 validly described species including both free-living and symbiotic forms. Members of this genus have been isolated from various marine sources, including water, sediments, fish, algae, marine animals and others. Because of their metabolic versatility and wide distribution in a variety of aquatic habitats, *Shewanella* and *Shewanella*-like organisms play a significant role in the cycling of organic carbon and other bionutrients.

The most of the structures of the carbohydrate antigens from these bacteria were reviewed earlier [10–12]. Below is a brief outline of the described structures.

*Shewanella oneidensis* is a Gram-negative bacterium associated with aquatic and subsurface environments [92]. It can attach to amorphous iron oxides and, in so doing, utilizes the Fe(II)/Fe(III) couple as a terminal electron acceptor during dissimilatory iron reduction.

Capsular polysaccharides (CPS) were extracted from *Shewanella oneidensis* strain MR-4, grown on two different culture media. The polysaccharides were analyzed using ^1^H and ^13^C NMR spectroscopy, and the following structure of the repeating unit was established (18) [93]:

(18) $$\begin{array}{l} \to 4)\text{-}\beta \text{-D-Man}p\text{-}(1\to 4)\text{-}\beta \text{-D-Glc}p\text{-}(1\to 4)\text{-}\beta \text{-D-Glc}p\text{NAc-}(\to \\ \alpha \text{-D-Quip}4\text{Nacyl-}(1\to 4)\text{-}\alpha \text{-D-Glc}p\text{A-}(1\to 3)\rfloor \end{array}$$

where the residue of 4-amino-4,6-dideoxy-d-glucose (Qui4N) was substituted with different *N*-acyl groups depending on the growth media. All monosaccharides are present in the pyranose form. In the PS from cells grown on enriched medium (trypticase soy broth, TSB) aerobically it was *N*-acylated with 3-hydroxy-3-methylbutyrate (60%) or with 3-hydroxybutyrate (40%), whereas in the PS from cells grown on minimal medium (CDM) aerobically it was acylated mostly with 3-hydroxybutyrate (>90%).

*Shewanella* spp. MR-4 produces a large quantity of CPS, which gives the cells “smooth” appearance; however, its LPS has no polymeric *O*-chain. The rough type LPS was analyzed using NMR, mass spectroscopy, and chemical methods. Two structural variants (I and II) have been found, both contained 8-amino-3,8-dideoxy-d-*manno*-octulosonic acid and lacked l-*glycero*-d-*manno*-heptose. A minor variant of the LPS contained phosphoramide substituent (19) [94]:

(19)
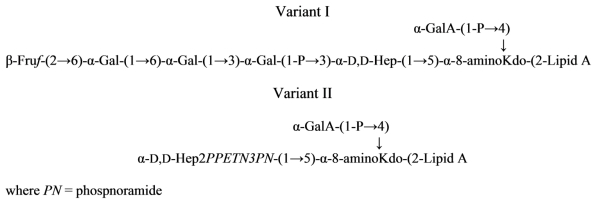


The structure of the *O*-specific polysaccharide from *Shewanella japonica* strain KMM 3601 has been elucidated. The initial and *O*-deacetylated lipopolysaccharides, and a trisaccharide representing the repeating unit, were studied by sugar analysis along with ^1^H and ^13^C NMR spectroscopy. The polysaccharide was found to contain a higher sugar, 5,7-diacetamido-3,5,7,9-tetradeoxy-d-*glycero*-d*talo*- non-2-ulosonic acid (a derivative of 4-epilegionaminic acid, 4-eLeg). The following structure of trisaccharide repeating unit was established (20) [95]:

(20) →4)-α-4eLeg5Ac7Ac-(2→4)-β-D-GlcpA3NAc-(1→3)-β-D-GalpNAc-(1→

This polysaccharide contains 5,7-di-*N*-acetyl derivative of the 4-eLeg which was found in nature for the first time. Earlier, the homopolymer of 5-*N*-acetamidoyl-7-*N*-acetyl-derivative of 4-eLeg was identified in the LPS of non-1 serogroup of *Legionella pneumophila* [96].

### 2.6. Genus *Aeromonas*

Species of the genus *Aeromonas* are common inhabitants of aquatic environments and have been described in connection with fish and human diseases [97–100]. This genus belongs to the family Aeromonadaceae [101,102] and, over the last two decades, the number of recognized species has expanded very rapidly.

A varied clinical picture of *Aeromonas* infections, including gastroenteritis, suggests complex pathogenic mechanisms. Strains of *Aeromonas hydrophila* serogroup O:34 are most common among mesophilic *Aeromonas* species [103], accounting for 26.4% of all isolates, and have been documented as an important cause of infections in humans [104].

The *O*-polysaccharide of *Aeromonas hydrophila* O:34 was obtained by mild-acid degradation of the lipopolysaccharide and studied by chemical methods and NMR spectroscopy before and after *O*-deacetylation. The polysaccharide was found to contain d-Man, d-GalNAc and 6-deoxy-l-talose (l-6dTal) (21) [105]:

(21)
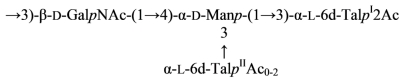


where 6dTal^I^ is *O*-acetylated stoichiometrically at position 2 and 6dTal^II^ carries no, one or two *O*-acetyl groups at any position. Although less common than l-rhamnose and l-fucose, 6-deoxy-l-talose occurs in a number of bacterial polysaccharides and is often present in an *O*-acetylated form. However, to the best of our knowledge, random *O*-acetylation has not been reported for either this or any other monosaccharide component of the lipopolysaccharides.

The core oligosaccharide of *A. hydrophila* (Chemotype III) lipopolysaccharide has been investigated. The studies involved the use of methylation analysis, oxidation with chromium trioxide, partial hydrolysis with acid, periodate oxidation, Smith degradation, and tagging of the reducing end-group. The core is unusual in having 3-acetamido-3,6-dideoxy-l-glucose as a constituent (22) [106]:

(22)
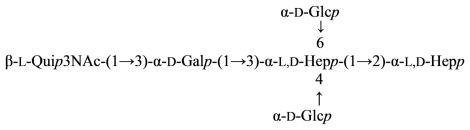


A rough strain of *Aeromonas hydrophila*, AH-901, has an *R*-type lipopolysaccharide with the complete core. The following core structure was established by chemical degradations followed by sugar and methylation analyses along with ESIMS and NMR spectroscopy (23) [107]:

(23)
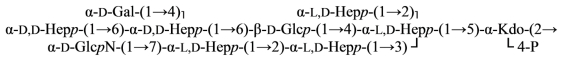


where α-d,d-Hep and α-l,d-Hep stand for d-*glycero*- and l-*glycero*-α-d-*manno*-heptose, respectively; Kdo stands for 3-deoxy-d-*manno*-oct-2-ulosonic acid; all monosaccharides are in the pyranose form; the degree of substitution with β-d-Gal is ~50%. Lipid A of the lipopolysaccharide has a 1,4′-bisphosphorylated β-d-GlcN-(1→6)-α-d-GlcN disaccharide backbone with both phosphate groups substituted with 4-amino-4-deoxyarabinose residues.

*A. salmonicida* is the aetiological agent of furunculosis in salmonid fish, a disease which causes high mortalities in aquaculture. Considerable effort has been devoted to the development of effective vaccines against furunculosis. Very little is known about the role of virulence factors *in vivo* and their role in furunculosis.

It was found, that when grown *in vitro*, *A. salmonicida* strain 80204-1 produced a capsular polysaccharide with the identical structure to that of the lipopolysaccharide *O*-chain polysaccharide. A combination of 1D and 2D NMR methods, including a series of 1D analogues of 3D experiments, together with capillary electrophoresis-electrospray MS (CE-ES-MS), compositional and methylation analyses and specific modifications was used to determine the structure of these polysaccharides. Both polymers were shown to be composed of linear trisaccharide repeating units consisting of 2-acetamido-2-deoxy-d-galacturonic acid (GalNAcA), 3-[(*N*-acetyl-l-alanyl)amido]-3,6-dideoxy-dglucose {3-[(*N*-acetyl-l-alanyl)amido]-3-deoxy-d-quinovose, Qui3NAlaNAc} and 2-aceacetamido- 2,6-dideoxy-d-glucose (2-acetamido-2-deoxy-d-quinovose, QuiNAc) as follows (24) [108]:

(24) →3)-α-D-GalpNAcA-(1→3)-β-D-QuipNAc-(1→4)-β-D-Quip3NAlaAc-(1→

where GalNAcA is partly presented as an amide and AlaNAc represents *N*-acetyl-l-alanyl group. CE-ES-MS analysis of CPS and *O*-chain polysaccharide confirmed that 40%of GalNAcA was present in the amide form. Direct CE-ES-MS/MS analysis of *in vivo* cultured cells confirmed the formation of a novel polysaccharide, a structure also formed *in vitro*, which was previously undetectable in bacterial cells grown within implants in fish, and in which GalNAcA was fully amidated.

To date, a limited number of bacteria have been reported to produce capsular and *O*-chain polysaccharides with identical structures. It appears that this property is not uncommon for fish pathogens and similar findings for *Listonella* (formerly *Vibrio*) *anguillarum* and *V. ordalii* [51,109] have previously been reported. It should be noted that the structures of the CPS and *O*-chain polysaccharide of *L. anguillarum* and *V. ordalii* have recently been re-examined and that the *galacto* configuration of the 2,3-diacetamido-2,3-dideoxy-hexuronic acid in both structures should be revised in favor of the *gulo* configuration.

The core oligosaccharide isolated from the lipopolysaccharide of *Aeromonas salmonicida* subsp. *salmonicida* has been investigated by methylation analysis, NMR spectroscopy (^13^C and ^1^H), oxidation with periodate and chromium trioxide, and Smith degradation (25) [110]:

(25)
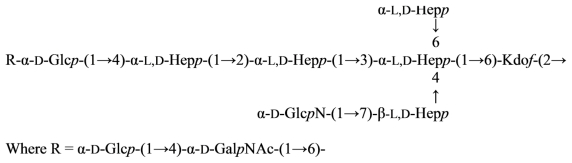


The core oligosaccharide structure of the *in vivo* derived rough phenotype of *Aeromonas salmonicida* subsp. *salmonicida* was investigated by a combination of compositional, methylation, CE-MS and one- and two-dimensional NMR analyses and established as the following (26) [111]:

(26)
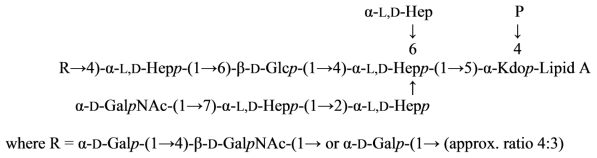


Comparative CE-MS analysis of *A. salmonicida* subsp. *salmonicida* core oligosaccharides from strains A449, 80204-1 and an *in vivo* rough isolate confirmed that the structure of the core oligosaccharide was conserved among different isolates of *A. salmonicida*.

While searching for bacteria that cross-react with the recently discovered second causative agent of cholera, *V. cholerae* O139 Bengal [112], six strains of another member of the family Vibrionaceae, *Aerornonas trota*, have been found to agglutinate with specific antiserum to *V. cholerae* O139 in a slide-agglutination test [113]: Polyclonal antiserum to a cross-reactive *A. trota* strain cross-protected infant mice against cholera on challenge with virulent *V. cholerae* O139. The cross-reactive bacteria were not serologically identical, and the antigenic relationship between them was of an a,b-a,c type, where a is the common antigenic epitope and b and c are unique epitopes. Serological and genetic studies suggested that the capsular polysaccharide has the same repeating unit as the *O*-antigen chain [114] and, thus, carries determinants of *O*-specificity.

The structure of the *V. cholerae* O139 capsular polysaccharide has been elucidated as (27) [115]:

(27)
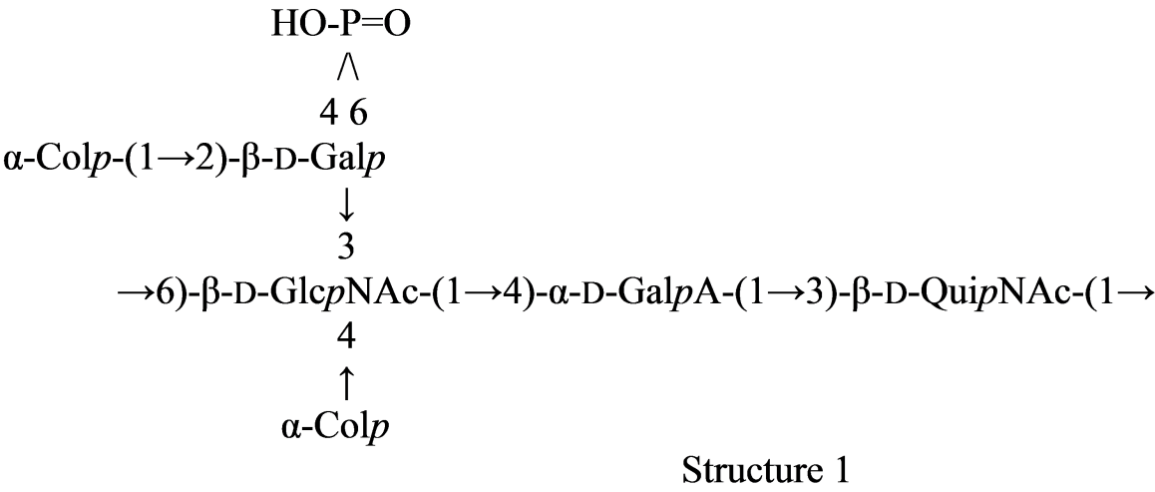


The presence of 3,6-dideoxy-l-*xylo*-hexose colitose (Col) and a cyclic phosphate diester are the unusual features of this polysaccharide.

The cross-reactive *A. trota* strains produce an *S*-type lipopolysaccharide with a polysaccharide *O*-antigen chain, which has not been chemically analyzed. To reveal a common epitope (or epitopes), which is responsible for the serological cross-reaction between these two microorganisms, the structure of the *O*-specific polysaccharide of *A. trota* strain 1354, which is one of the six cross-reactive strains was determined. On the basis of methylation analysis and NMR spectroscopic studies of the initial and modified, colitose-free polysaccharide, it was concluded that the repeating unit of the *O*-specific polysaccharide has the following structure (28) [116]:

(28)
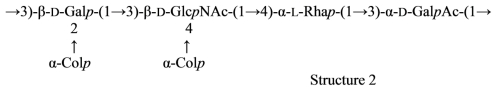


Mild hydrolysis of the polysaccharide with 48% hydrofluoric acid at 4 °C removed the Col residues completely without significant depolymerization. As a result, a modified polysaccharide was obtained, which lacked colitose but contained the four other sugar constituents of the repeating unit (29):

(29) $$\begin{array}{c} \to 3)\text{-}\beta \text{-D-Gal}p\text{-}(1\to 3)\text{-}\beta \text{-D-Glc}p\text{NAc-}(1\to 4)\text{-}\alpha \text{-L-Rha}p\text{-}(1\to 3)\text{-}\alpha \text{-D-Gal}p\text{Ac-}(1\to \\ \text{Structure}\;3 \end{array}$$

Although structurally different, the repeating unit 2 of the *A. trota O*-specific polysaccharide has a tetrasaccharide fragment in common with the repeating unit 1 of the capsular polysaccharide of cross-reactive *V. cholerae* O139, which includes Gal, GlcNAc, and two terminal Col residues. It seems likely that the common antigenic epitope is associated with the non-reducing terminal end of the polysaccharide, as was suggested for the oligosaccharide epitopes that determine the blood-group activities of some *Salmonella* and *Escherichia coli* strains [117,118]. Thus, structure 1 corresponds to the biological repeating unit of the *V. cholerae* O139 polysaccharide, and, therefore, its non-reducing terminal tetrasaccharide fragment has the structure 4. With an assumption that the structure 2 also corresponds to the biological repeating unit of the *A. trota* polysaccharide, its non-reducing terminal end should be occupied by the tetrasaccharide fragment 5, which differs from the tetrasaccharide 4 in lacking the cyclic phosphate group only (30).

(30)
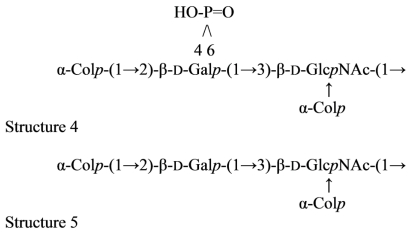


The known cross-reactivity between the strain studied and *Vibrio cholerae* O139 Bengal is substantiated by the presence of a common colitose-containing epitope shared by the *O*-specific polysaccharide of *A. trota* and the capsular polysaccharide of *V.cholerae*, which is thought to carry determinants of *O*-specificity.

*Aeromonas bestiarum* is a member of the bacterial species belonging to the motile aeromonad group that comprises several species such as *A. hydrophila*, *A. sobria*, *A. veronii*, *A. allosaccharophila* and *A. jandaei*, which have been reported as fish pathogens. [119,120]. The results of taxonomic studies revealed that diseases, and thus losses, in commercial carp were mostly caused by strains identified as *A. bestiarum*. However, until now there has been limited knowledge of the compositional diversity of the *O*-antigenic part of LPS among *Aeromonas* species. Therefore, it appeared purposeful to undertake immunochemical studies of *Aeromonas* strains with the described genomospecies (16S rDNA-RFLP) and their pathogenicity, which then could complete the LPS-based classification data of *Aeromonas* strains. Recently published structural studies of the *R*-type LPS from *A. hydrophila* strain AH-901 have extended this database [107]. The LPS of the transposone mutant AH-901 of the wild-type strain *A. hydrophila* AH-3 was devoid of the *O*-chain polysaccharide and contained a complete core with heptoses as the most dominant sugar residues and a lipid A with a diglucosaminyl backbone containing two phosphate groups substituted with 4-amino-4-deoxyarabinoses. Still, the question remains how common this structure of the core region is among motile and non-motile *Aeromonas* species.

The *O*-specific polysaccharide obtained by mild-acid degradation of *A. bestiarum* 207 lipopolysaccharide was studied by sugar and methylation analyses along with ^1^H and ^13^C NMR spectroscopy. The sequence of the sugar residues was determined by ROESY and HMBC experiments. It is concluded that the *O*-polysaccharide is composed of branched pentasaccharide repeating units (31) [121]:

(31)
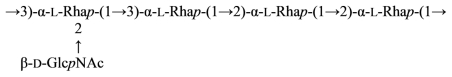


The determined structure is different from those published for other *Aeromonas* strains. However, the α-l-Rha residue was found in the *O*-chain polysaccharides of *A. cavia*e strains 11212 and ATCC 15468, as one of the components of their pentasaccharide and tetrasaccharide repeating units, respectively [122,123]. Another 6-deoxyhexose residue, 6-deoxy-l-talose, was identified as a dominant component of the *A. hydrophila* O:34 OPS. Interestingly, strains of that serogroup are most common among mesophilic *Aeromonas* species, accounting for 26.4% of all isolates, and have been documented as an important cause of human infections. It is unknown whether this group of isolates is homogeneous with respect to their OPS composition. If some departures from the typical *O*-antigen structure were found, this could suggest the presence of an immunochemical heterogeneity of the isolates, similar to that observed among *A. salmonicida* strains [105].

*Aeromonas caviae* is associated with gastrointestinal disease in adults and acute, severe gastroenteritis in children [124]. A number of putative virulence factors have been identified for *A. caviae*, including polar flagella, pili and cytotoxin. Both flagella and pili have been implicated in the adherence of *A. caviae* to human epithelial cells *in vitro*. Some strains of *A. caviae* are able to form biofilms on inert surfaces, a phenomenon attributed to a hyperpiliation of the cells through type IV pili [125]. Less is known about the role of LPS in these processes, although the *flmA* and *flmB* genes of *A. caviae* Sch3N have been implicated in LPS *O*-chain biosynthesis [126]. As a major cell-surface component, LPS of *A. caviae* has been implicated in the adherence to human epithelial cells and biofilm formation, but the role of LPS in the pathogenesis of *A. caviae*-induced gastroenteritis is not well understood. Comprehensive structural and genetic studies of LPS are essential to determine its etiological role in pathology of the disease.

*A. caviae* strain 11212 was isolated from the stools of a patient with diarrhea. Sugar analysis, methylation analyses, and a uronic acid degradation together with NMR spectroscopy were the principal methods used in the structural study of the *O*-polysaccharide from the LPS of this strain. The sequence of the sugar residues could be determined by NOESY and HMBC experiments. It is concluded that the polysaccharide is composed of pentasaccharide repeating units (32) [123]:

(32)
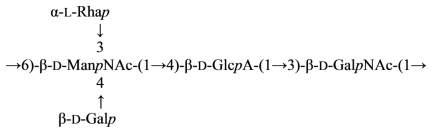


The *O*-chain polysaccharide produced by a mild acid degradation of *A. caviae* ATCC 15468 lipopolysaccharide was found to be composed of l-rhamnose, 2-acetamido-2-deoxy-d-glucose, 2-acetamido-2-deoxy-d-galactose and phosphoglycerol. Subsequent methylation and CE–ESIMS analyses and 1D/2D NMR (^1^H, ^13^C and ^31^P) spectroscopy showed that the *O*-chain polysaccharide is a high-molecular-mass acidic branched polymer of tetrasaccharide repeating units with a phosphoglycerol substituent having the following (33) [127]:

(33)
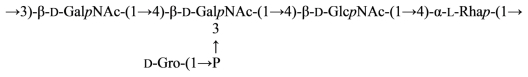


The previously determined structure of the *O*-chain polysaccharide from *A. caviae* strain 11212 bears no resemblance to the above structure, suggesting the possible need to divide this species into more than one serological group.

Interestingly, the phosphoglycerol moiety identified in the structure of the *O*-chain polysaccharide of *A. caviae* ATCC 15468 was previously found in the *O*-chain polysaccharides of *Citrobacter* 016 [128], *Hafnia alvei* strain PCM1207 [129] and *Proteus* species [130], as well as in the exo-polysaccharide produced by *Lactobacillus sake O*-1 and the specific capsular polysaccharide of *Streptococcus pneumoniae* type 45 [131]. It is recognized as an immunodominant epitope, and the cross-reactions between the LPS of *Citrobacter* O16 and *H. alvei* strain PCM 1207 could be attributed to the presence of this shared epitope in their respective *O*-specific polysaccharide structures.

At present, much research on *Aeromonas* bacteria is focused in epidemiology [132] and immunology [133]. However, since the polysaccharides obtained from many different bacteria are important in the manufacture, distribution, storage and consumption of food products [134], cosmetics and paints, *Aeromonas* polysaccharides are also receiving attention for similar applications.

The *Aeromonas nichidenii* strain 5797 produces an acidic polysaccharide—*Aeromonas* gum. This gum exists as aggregates in aqueous solutions and exhibits a very high viscositythis form. In addition, the gum exists in semi-flexible single chains in cadoxen and dimethyl sulfoxide solutions at room temperature. Structural analysis of this polysaccharide may provide a basis for a better understanding of its use as a gelling agent for food products and for other uses.

This gum was studied by ^1^H and ^13^C NMR spectroscopy including 2D COSY, TOCSY, HMQC, HMBC and ROESY experiments after *O*-deacetylation and Smith degradation. These investigations revealed the presence of an *O*-acetylated pentasaccharide repeating unit composed of mannose, glucose, xylose and glucuronic acid, and having the following structure (34) [135]:

(34)
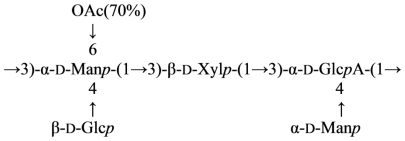


After the *O*-deacetylation with following Smith degradation of the native gum, the modified polysaccharide building up of linear trisaccharide repeating unit (35) was obtained:

(35) →3)-α-D-Manp-(1→3)-β-D-Xylp-(1→3)-α-D-GlcpA-(1→

Other bacterial polysaccharide gums used in the food industry are xantan gum from *Xantomonas campestris* [136], alginates from *Pseudomonas aeruginosa* [137] and *Azobacter vinelandii* and gellan gum from *Sphingomonas paucimobilis* [138]. Acetylation at position 6 of mannose and the presence of glucose snd glucuronic acid are the only structural similarities of the xantan and aeromonas gums. Gellan gum also has the sugars glucose and glucuronic acid, although the *O*-acetylation at position 6 is on a glucose residue [139].

### 2.7. Genus *Idiomarina*

The bacterial strain KMM 231^T^ was isolated from a seawater sample taken at a depth of 4000 m from the northwestern Pacific Ocean. This deep-sea strain was found to be Gram-negative, halotrophic, psychrotolerant, heterotrophic and strictly aerobic. On the basis of polyphasic evidence, it was proposed that strain KMM 231 be classified in the new genus, *Idiomarina* gen. nov., as a representative of *Idiomarina zobellii* sp. nov. [140].

The *O*-polysaccharide was obtained by mild base degradation of the lipopolysaccharide. The following structure of the *O*-polysaccharide was elucidated by ^1^H and ^13^C NMR spectroscopy of the oligosaccharide and base-degraded lipopolysaccharide, including COSY, TOCSY, ROESY, ^1^H, ^13^C HSQC, HSQC-TOCSY and HMBC experiments (36) [141]:

(36) $$\begin{array}{c} \to 3)\text{-}\alpha \text{-D-Qui}p4\text{N-}(1\to 4)\text{-}\alpha \text{-D-Glc}p\text{A-}(1\to 6)\text{-}\alpha \text{-D-Glc}p\text{NAc-}(1\to 4)\text{-}\alpha \text{-L-Gulc}p\text{NA-}(1\to 3)\text{-}\alpha \text{-D-}\\ \text{Fuc}p\text{NAc-}(1\to \end{array}$$

Mild acid degradation of the lipopolysaccharide yielded an oligosaccharide, which represents one repeating unit of the *O*-polysaccharide (37):

(37) $$\begin{array}{c} \alpha \text{-D-Qui}p4\text{N-}(1\to 4)\text{-}\alpha \text{-D-Glc}p\text{A-}(1\to 6)\text{-}\alpha \text{-D-Glc}p\text{NAc-}(1\to 4)\text{-}\alpha \text{-L-Gulc}p\text{NA-}(1\to 3)\text{-}\alpha \text{-D-}\\ \text{Fuc}p\text{NAc} \end{array}$$

The *O*-polysaccharide is distinguished by the presence of two unusual amino sugars, 4-amino-4,6-dideoxy-d-glucose (d-Qui4N) and 2-amino-2-deoxy-l-guluronic acid (l-GulNA), both having the free amino group. The unexpectedly high acid lability of the glycosidic linkage of 2-acetamido-2,6-dideoxy-d-galactose (d-FucNAc) could be associated with the presence of a free amino group adjacent to the site of attachment of FucNAc to Qui4N. 2-amino-2-deoxy-l-guluronic acid, which has been previously found in nature only in a few bacterial glycopolymers, including the acidic capsular polysaccharides of *Vibrio parahaemolyticus* K15 [142] and the marine bacteria bacteria *Pseudoalteromonas nigrifaciens* KMM 158 [32] and KMM 161 [143], as well as in the cell wall of *Halococcus* sp. strain 24 [144]. Another rare component of the *O*-polysaccharide, 4-amino-4,6-dideoxy-d-glucose, has not been discovered previously with the free amino group but rather carrying various *N*-acyl substituents, including formyl [145], acetyl [146], *N*-acetylglycyl [147], *N*-[(*R*)-3-hydroxybutyryl]-l-alanyl [148] and other groups.

## 3. Structure of Carbohydrate Antigens of *Cytophaga-Flavobacterium-Flexibacter* Phylum

### 3.1. Genus *Flexibacter*

The *Cytophaga-Flavobacterium-Flexibacter* bacteria are represented by a large, somewhat heterogeneous group of filamentous, gliding, Gram-negative bacteria with unusual surface properties [149]. At least seven members of this group are considered to be important fish pathogens. They infect a wide variety of fish species and usually form biofilms, primarily on the tissues associated with the oral cavity. Among these, *Flexibacter maritimus* has recently emerged as a cause of widespread severe mortality and economic losses in farmed marine species worldwide [150].

*Flexibacter maritimus*, a long rod-shaped, Gram-negative bacterium, has been associated with disease (Flexibacteriosis) in a number of fish species [151] and its economic importance has been related to a cause of cutaneous erosion disease particularly in sea-caged salmonids. In grouper, *Flexibacter maritimus* causes “red boil” disease [152] related to its clinical signs of reduced scales and severe hemorrhage on the body surface, resembling boiled skin and causing a high mortality rate. No effective vaccine has been developed against this pathogen. A clearer definition of the relevant immunoreactive macromolecules of these bacterial fish pathogens is fundamentally important particularly with regard to the mechanisms of pathogenesis and the role of infective biofilms. This information is important for the development of appropriate immunochemical reagents to facilitate speciation and the design of cost-effective, efficacious vaccines. Lipopolysaccharides (LPS, endotoxins) play a role in the pathogenesis of Gram-negative infections and the structural analysis of their antigenic *O*-polysaccharide (*O*-PS) components is important in providing a molecular level understanding of their serological specificities, role in pathogenesis, development of diagnostic agents, and the production of *O*-PS based conjugate vaccines.

**Figure 2:**
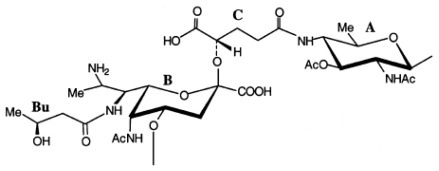
LPS structure of *Flexibacter maritimus* [153]. Reproduced with permission from Wiley-Blackwell.

An acidic *O*-specific polysaccharide, obtained by mild acid degradation of the *Flexibacter maritimus* LPS was found to be composed of a disaccharide repeating unit built of 2-acetamido-3-*O*-acetyl-4- [(*S*)-2-hydroxyglutar-5-ylamido]-2,4,6-trideoxy-β-glucose and 5-acetamido-7-[(*S*)-3-hydroxybutyramido]-8-amino-3,5,7,8,9-pentadeoxynonulopyranosonic acid (Sug) having the structure (Figure 2) [153].

This *O*-PS contained a new nonulosonic acid derivative, 5-(3-hydroxybutyramido)-7-acetamido-8- amino-3,5,7,8,9-pentadeoxy-β-*manno*-nonulopyranosonic acid, with as yet undetermined configuration at C-8 and tentatively assigned the l-absolute configuration. Moreover, it contains a linkage involving a *R*-2-hydroxyglutaric acid residue reported for the first time as a bacterial polysaccharide component. A similar component, *O*-glycosylated amide linked *R*-malic acid was reported as a component of the *O*-PS from another fish pathogen *Flavobacterium psychrophilum* [154].

### 3.2. Genus *Flavobacterium*

The genus *Flavobacterium* includes more than 60 validly described species, isolated from various sources [155]. The pathogenesis of infection with *Flavobacterium* spp. is not well understood; in humans, however, they cause neonatal meningitis, catheter-associated bacteremia, and pneumonia, and have also been associated with some cases of HIV disease [156]. *Flavobacterium* spp. are also characterized by an unusual pattern of antibiotic sensitivity, being resistant to several antimicrobials effective against Gram-negative rods.

*Flavobacterium psychrophilum* (syn. *Cytophaga psychrophilia*, *Flexibacter psychrophilus*) is the etiological agent of rainbow trout fry syndrome (RTFS) and bacterial cold water disease, septicemic infections that can cause significant early losses in hatchery-reared salmonids, particularly Rainbow trout (*Oncorhynchus mykiss*) in Europe, and coho salmon (*Oncorhynchus kisutch*) in North America. In the past decade, *Flexibacter psychrophilum* has emerged as a causative agent of severe rainbow trout fry mortality in Europe (RTFS) and is now known to affect salmonids worldwide [157]. The molecular pathogenesis of *Flexibacter psychrophilum* is primarily limited to their exotoxins and plasmids [158]. No vaccine is commercially available for RTFS control and the development of effective, inexpensive, easily administered vaccines and specific diagnostics have become an important goal to reduce losses that occur in immature salmonids. Following a study to differentiate *Flexibacter psychrophilum* from other closely related bacteria using both randomly amplified polymorphic (RAPD)-PCR and polyclonal antibodies, several immunogenic cell surface molecules, including lipopolysaccharides (LPS) that may be involved in pathogenesis were identified as potential vaccine candidates [159]. Recently, several *Flexibacter psychrophilum* surface molecules, including lipopolysaccharide (LPS), have been implicated in its pathogenesis and identified as potential vaccine and diagnostic candidate macromolecules.

The structure of the antigenic *O*-polysaccharide contained in the LPS of *Flexibacter psychrophilum* strain 259-93 was deduced by the application of analytical NMR spectroscopy, mass spectrometry, glycose and methylation analysis, and partial hydrolysis degradations, and was found to be an unbranched polymer of trisaccharide repeating units composed of l-rhamnose (l-Rha*p*), 2-acetamido- 2-deoxy-l-fucose (l-Fuc*p*NAc) and 2-acetamido-4-((3*S*,5*S*)-3,5-dihydroxyhexanamido)-2,4-dideoxy- d-quinovose (d-Qui*p*2NAc4NR, 2-*N*-acetyl-4-*N*-((3*S*,5*S*)-3,5-dihydroxyhexanoyl)-d-bacillosamine) (1:1:1) (38) [154]:

(38) $$\begin{array}{l} \to 4)\text{-}\alpha \text{-L-Fuc}p\text{NAc-}(1\to 3)\text{-}\alpha \text{-D-Quip}2\text{NAc}4\text{NR-}(1\to 2)\text{-}\alpha \text{-L-Rha}p\text{-}(1\to \\ \text{where}\;\text{R}\;\text{is}\;(3\text{S},5\text{S}){\text{-CH}}_{3}\text{-CH}(\text{OH}){\text{-CH}}_{2}\text{-CH}(\text{OH})\text{-CO-} \end{array}$$

The occurrence of *N*-acyl derivatives of 2,4-diamino-2,4-dideoxy-d-quinovose (bacillosamine) in bacterial glycans is not unusual. The glycose has been demonstrated to be a constituent of the *O*-antigens of *Fusobacterium necrophorum* [160], *Pseudomonas aurantiaca* IMB 31 [161], *Vibrio cholerae* O:3 and O:5 [162,163], *Pseudomonas aeruginosa* [164], and also in the capsular polysaccharide of *Alteromonas* sp. CMM 155 [31] and a polysaccharide component of *Bacillus licheniformis* [165]. It is of interest that the parent 2,4-diamino-2,4-dideoxy-d-quinovose residue present in the backbone chain of the *O*-PS of *V. cholerae* O:3 LPS [162], shown below, was acylated at the amino group at C-4 by a 3,5-dihydroxyhexanoyl group of undetermined configuration. (3*S*,5*S*)-Dihydroxyhexanoic acid has been found in berries of *Sorbus aucuparia* as the β-d-glucopyranoside of its d-lactone [166]. The 3,5-dihydroxyhexanoic acid in the *O*-PS of *Flexibacter psychrophilum* has the same configuration.

*Flavobacterium columnare*, formerly referred to as *Flexibacter columnaris* or *Cytophaga columnaris* [155], is a Gram-negative bacterium which causes columnaris disease in warm water fish, a disease that is the second leading cause of mortality in pond raised catfish in the south-eastern United States.

The virulence factors of *Flavobacterium columnare* are relatively unknown, but it has been suggested that, in pathogenesis, adhesion of the bacterium may be related to its surface polysaccharide constituents [167]. This investigation was directed towards characterization of the lipopolysaccharide (LPS) and putative capsule produced by the bacterium, as a first step in identifying their possible role in pathogenesis in fish. In addition, it was considered that characterization of the LPS *O*-polysaccharide (*O*-PS) antigen would provide a structural knowledge basis for the development of a specific antibody diagnostic agent and possible target molecules for a conjugate based vaccine.

The structure of the antigenic *O*-chain polysaccharide of *Flavobacterium columnare* ATCC 43622, a Gram-negative bacterium that causes columnaris disease in warmwater fish, was determined by high-field 1D and 2D NMR techniques, MS, and chemical analyses. The *O*-chain was shown to be an unbranched linear polymer of a trisaccharide repeating unit composed of 2-acetamido-2-deoxy-dglucuronic acid (d-GlcNAcA), 2-acetamidino-2,6-dideoxy-l-galactose (l-FucNAm) and 2-acetamido- 2,6-dideoxy-d-*xylo*-hexos-4-ulose (d-Sug) (1:1:1) (39) [52]:

(39)
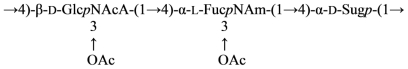


It is interesting to note that O3-linked d-Sug was found to be a component of the *O*-PS of the fish pathogen *Vibrio ordalii* serotype O:2 [113], which is the cause of vibriosis among feral and farmed fish and shellfish. The only other reported bacterial source of this glycose is the specific CPS of *Streptococcus pneumoniae* type 5 [50]. However, in the latter polysaccharides, the glycose is found in its β-d-configuration in contrast with the α-d-configuration found in the *F. columnare O*-PS. In agreement with previous studies, we also found that the presence of this 4-ketoglycose in the polymeric structure rendered the *O*-PS unstable under alkaline conditions and even prolonged storage in aqueous solutions at pH 7. A similar result was found in a study of forbeside C, a saponin of *Asterias forbesi* [55], which also has a component d-Sug residue.

### 3.3. Genus *Cellulophaga*

The genus *Cellulophaga* belongs to the family Flavobacteriaceae of the phylum Bacteroidetes. It was created to accommodate the heterotrophic aerobic Gram-negative yellow/orange pigmented gliding and agarolytic bacteria of marine origin. Currently this genus comprises six validly described species: *C. algicola*, *C. baltica*, *C. fucicola*, *C. lytica*, *C. pacifica* and *C. tyrosinoxydans* [19]. Data on the *O*-polysaccharide structure of *Cellulophaga* were reported only for *C. baltica* [168] and *C. fucicola* [169] strain NN015860^T^ isolated from brown alga *Fucus serratus* L., which inhabits the North Sea, Atlantic Ocean.

The *O*-polysaccharide was isolated from the lipopolysaccharide of *Cellulophaga fucicola* and studied by chemical analyses along with ^1^H and ^13^C NMR spectroscopy. The following new structure of the *O*-polysaccharide of *C. fucicola* containing 5,7-diacetamido-3,5,7,9-tetradeoxy-l-*glycero*-l*manno*- non-2-ulosonic acid residue (pseudaminic acid, Pse*p*) was elucidated as the following (40):

(40) →4)-β-D-Galp-(1→4)-β-D-Glcp-(1→4)-β-Pse-(2→

From the chemical and NMR spectroscopy data it was shown that the repeating unit of this PS from the LPS of *C. baltica* constructed in tetrasaccaride units, containing two d-mannose, 2-acetamido-2-deoxy-d-glucose and d-glucuronic acid residues, and non-stoichiometric quantity of the *O*-acetyl groups (41):

(41)
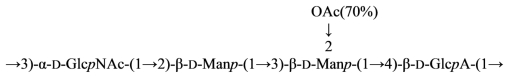


The influence of the *O*-acetyl group on the distal sugar residues in the repeating unit was shown.

## 4. Structure of Carbohydrate Antigens of *Alphaproteobacteria*

### 4.1. Genus *Sulfitobacter*

The genus *Sulfitobacter* was established in 1995 for marine gram-negative heterotrophic bacteria isolated from the H_2_S/O_2_ zone of the Black Sea and includes 10 validly described species. These microorganisms belongs phylogenetically to the cluster *Roseobacter-Ruegeria-Sulfitobacter* of class *Alphaproteobacteria* [19].

The microorganism *Sulfitobacter brevis* KMM 6006 was isolated from the bottom sediments in Chazma Bay (Sea of Japan). The glycopolymer was isolated from this strain was obtained and found to be teichoic acid containing ribitol, glycerine, and *N*-acetyl-d-glucosamine. The polymeric chain was built up of alternating 1,5-poly(4-*N*-acetyl-β-d-glucosaminylribitophosphate) and 1,3-poly (glycerophosphate) based on ^13^C and ^31^P NMR spectroscopy of the native polymer and the glycoside obtained by its dephosphorylation (42) [170]:

(42)
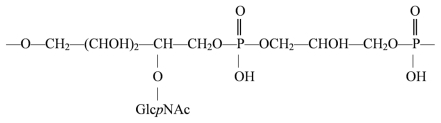


### 4.2. Genus *Loktanella*

*Loktanella rosea*, a marine Gram-negative bacterium isolated from sediments of Chazma Bay, Sea of Japan [171]. *L. rosea* is a species nova that belongs to the genus *Loktanella*, which was created in 2004 in order to classify some new heterotrophic Alphaproteobacteria collected from Antartic lakes. *L. rosea* is a mesophilic and chemo-organotroph bacterium with a respiratory metabolism whose growth needs a medium with 1–12% of NaCl.

**Figure 3:**
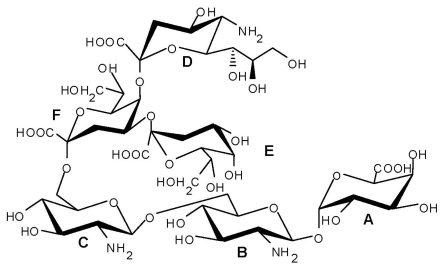
Carbohydrate structure of *Loktanella rosea* [172]. Reproduced with permission from Oxford Univeristy Press.

The LPS from *L. rosea* has been defined through sugar analysis, 2D nuclear magnetic resonance (NMR) and matrix-assisted laser desorption ionization (MALDI) mass spectrometry investigation. A unique highly negatively charged carbohydrate backbone has been identified. The lipid A skeleton lacks the typical phosphate groups and is characterized by two β-GlcNs and an α-galacturonic acid (GalA). This was the first example of a lipid A saccharide backbone in which the α-GlcN-phosphate residue is replaced by a β-GlcN-(1→1)-α-GalA in a mixed trehaloselike linkage (Figure 3).

The core region is built up of three ulosonic acids, with two 3-deoxy-d-*manno*-oct-2-ulosonic acid residues, one of which is carrying a neuraminic acid (Neu). The overall carbohydrate structure is an exceptional variation from the typical architectural skeleton of endotoxins and also implies a very different biosynthesis (43) [172].

(43)
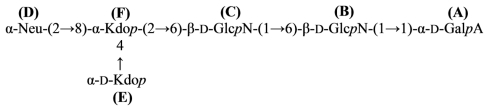


The lipooligosaccharide (LOS) was found to be characterized by a novel and unique hexasaccharide skeleton comprising: (i) a very small core region exclusively composed of ulosonic sugars and containing an Neu attached to a Kdo unit: α-Neu-(2→8)[α-d-Kdo-(2→4)]-α-d-Kdo-(2→ and; (ii) an exceptional lipid A backbone: β-d-GlcN-(1→6)-β-d-GlcN-(1→1)-α-d-GalA in which both GlcN residues were present with a β-anomeric configuration. Moreover, it lacked the classical phosphate residues at *O*-4′ and *O*-1, this latter was replaced by an α-GalA linked in a mixed trehalose-like linkage. To the best of our knowledge, this kind of glycosydic linkage was never found in biomolecules; its presence obviously implies profound biosynthetic differences from the canonical LPS lipid A pathway [173].

## 5. Conclusions

There is an extensive diversity between the chemical structures of the carbohydrate antigens from the different marine bacteria reviewed here. These polymers provide a rich source of uncommon monosaccharides, including higher sugars, and their derivatives having various non-sugar substituents.

In contrast to all LPS structures known to date, LPSs of the *Shewanella* genus contain a novel linking unit between the core polysaccharide and lipid A moieties, namely 8-amino-3,8-dideoxy-d*manno*- octulosonic acid (Kdo8N). The lipooligosaccharide (LOS) from *Loktanella rosea* is characterized by a novel and unique hexasaccharide skeleton among the known LOS structures.

This chemical structural information of carbohydrate-containing biopolymers may be useful in classification of Gram-negative marine bacteria and elaborating the current concepts regarding the organization and mechanisms of functioning of their cell wall.

## Subject Index

Kdo-3-deoxy-d-*manno*-oct-2-ulosonic acid [4]

l,d-Hep-l-*glycero*-d-*manno*-heptose [4]

Kdo8N-8-amino-3,8-dideoxy-d-*manno*-oct-2-ulosonic acid [14–16]

d,d-Hep-d-*glycero*-d-*manno*-heptose [15,16]

d-Gal*p*2NAcA6(d-Ala)-2-acetamido-2-deoxy-*N*-(d-galacturonyl)-d-alanine [27]

d-Man*p*NAcA-2-acetamido-2-deoxy-d-mannuronic acid [41]

d-Fuc*p*NThrAc-2,6-dideoxy-2-(*N*-acetyl-l-threonine)amino-d-galactose [41]

[2-deoxy-(*N*-acetyl-l-threonine)amino-d-fucose] [41]

l-Gal*p*NAm3AcA-2-acetamidino-3-acetamido-2,3-dideoxy-l-galacturonic acid [48]

Sug-2-acetamido-2,6-dideoxy-d-*xylo*-hexos-4-ulose [48]

d-Glc*p*NAcylA-2-acetamido-3-(*N*-malyl)amino-2,3-dideoxy-d-glucuronic acid [48]

l-Glc*p*NmalylA-2-acetamido-3-(*N*-malyl)amino-2,3-dideoxy-l-glucuronic acid [52]

d-Sug-2-acetamido-2,6-dideoxy-d-*xylo*-hexos-4-ulose [52]

d-Glc*p*NAc3NacylAN-2-acetamido-3-acylamino-2,3-dideoxy-d-glucuronamide [53]

4-d-malyl (~30%) or 2-*O*-acetyl-4-d-malyl (~70%) [53]

l-Gal*p*NAmA-2-acetimidoylamino-2-deoxy-l-galacturonic acid [53]

d-Qui*p*NAc-2-acetamido-2,6-dideoxy-d-glucose [53]

d-GlcN3NA-2,3-diamino-2,3-dideoxy-d-glucuronic acid [54]

6d-β-d-Hep*p*-6-deoxy-β-d-*manno*-heptopyranose [71]

d-Qui3NAcyl-3-amino-3,6-dideoxy-d-glucose acylated with 3-hydroxy-2,3-dimethyl-5- oxopyrrolidine- 2-carboxylic acid [72]

l-PneNAc4OAc-2-acetamido-4-*O*-acetyl-2,6-dideoxy-l-talose [73]

Hb-(*S*)-3-hydroxybutanoyl [73]

d-Glc*p*-NAc3NRA-2-acetamido-3-[(d-3-hydroxybutyl)]amido-2,3-dideoxy-d-glucuronic acid [74]

l-Fuc*p*Am3OAc-2-acetimidoylamino-3-*O*-acetyl-2,3-dideoxy-l-fucose [74]

l-AltNAcA-2-acetamido-2-deoxy-l-altruronic acid [85]

4eLeg5Ac7Ac-5,7-diacetamido-3,5,7,9-tetradeoxy-d-*glycero*-d-*talo*-non-2-ulosonic acid [95]

d-Qui3NAlaNAc-3-[(*N*-acetyl-l-alanyl)amido]-3,6-dideoxy-d-glucose [108]

Col-3,6-dideoxy-l-*xylo*-hexose (colitose) [115]

d-Qui4N-4-amino-4,6-dideoxy-d-glucose [141]

l-GulNA-2-amino-2-deoxy-l-guluronic acid [141]

d-Qui2NAc4NR-2-acetamido-4-((3*S*,5*S*)-3,5-dihydroxyhexanamido)-2,4-dideoxy-d-quinovose [154]

Pse (pseudaminic acid)-5,7-diacetamido-3,5,7,9-tetradeoxy-l-*glycero*-l-*manno*-non-2-ulosonic acid [169]

Neu (neuraminic acid)-5-amino-3,5-dideoxy-d-*glycero*-d-*galacto*-non-2-ulosonic acid [172]
